# Jefferson Medical College

**Published:** 1844-06-15

**Authors:** Edward R. Squibb


### CLINICAL LECTURES AND REPORTS.

### JEFFERSON MEDICAL COLLEGE.

*April* 24, 1844.

#### CLINIC OF PROFESSOR MÜTTER.

[Reported by Edward R. Squibb.]

The first case which presents this morning, is one of great interest and importance. This patient, as may be seen, has a very extensive tumour occupying the whole side of the neck, from the lower jaw to the clavicle; the location of which at once indicates its importance, involving, as it does, many of the vital parts. First made its appearance when the patient was only 3 years old, when the attendant, supposing it to be a scrofulous enlargement of a lymphatic gland, applied to it a powerful escharotic, which, fortunately for the patient, however, was not sufficiently active to effect his purpose. Continuing to augment in size, it at present fills the whole space indicated; causing the patient to incline her head to the opposite side. Upon examination we find a hardened mass, which being quite movable, we should judge to be external to, and independent of the deep seated facia; we find something more too, —there is evident pulsation in the tumour, which may be seen distinctly in the light in which she sits. What does this indicate? Not necessarily an aneurism, and this is important to be borne in mind. A simple fatty tumour might be found to pulsate from mere contiguity to some large artery. How then shall we make the diagnosis? Simply by raising it as much as possible from the situation which it occupies when the pulsation is felt; which, if continuing unabated, would lead to the inference of an aneurismal sack, which appears to exist in the case before us,—the hardness of the tumour being probably due to the accumulation of clots which are usually found to occupy the outer portions of the sack.

As these cases always give rise to serious consequences, the prognosis, from their very nature, must be unfavourable.

What is to be done in such cases? The ligature of the main artery by the Hunterian operation, if possible, is the only means which may be used with any prospect of success, and therefore the proper one in this case. Although the ligature of this large trunk may, for a time, modify the condition of the brain, yet it has not been found to implicate its functions to any extent in the number of cases upon which it has been performed; even where it has been tied on both sides of the same patient, it has not materially interfered with innervation, provided time was allowed between the operations, for the anastomosing branches to accommodate themselves to their increase of function.

Case II.—Mrs. M.'s child æt. 2. This is a case of Prolapsus Ani, or as it is commonly termed among the people "falling down of the body," and occurred first, as the mother informs us, when the child was only 3 days old. It is one of the most terrible cases of this affection that I have ever seen; the sphincter muscle appears to be entirely powerless, permitting when pressure is taken off, an immense mass of intestine to protrude, as you see, on the least movement of the child, and throwing it into spasms, from the irritation produced.

Can this be remedied in any way? How shall we cure it? The operation which I prefer, and the one upon which most confidence is placed, among the many which have been proposed, is that first suggested by Hey, but which was afterwards employed by, and attributed to Dupuytren, which consists in removing by means of scissors and forceps, some of the radiating folds of skin, which surround the orifice of the rectum. In this case it will be necessary to remove five or six of these folds,—in those of ordinary occurrence, three or four will be found sufficient. This is effected as you see by pinching up a fold in the forceps, and then cutting it off with a simple clip with the scissors. When cicatrisation takes place in these slight wounds, the orifice becomes contracted, thereby preventing a further protrusion. We shall order this child a T bandage, to be applied to the part firmly, in order that no prolapsion may occur during the healing, and also that the child shall be kept under the effect of opium to prevent the recurrence of the spasms. In case the child was a little older, we might, by an entire change of diet, so modify the nutrition of the system, as to produce a radical change in the nervous and muscular power, and thereby aid ourselves much in the treatment of this most distressing complaint.

Case III.—Is one of common every day occurrence, and therefore entitled to some attention, viz: Polypus of the nose. By polypus is meant any tumour attached by a narrow base or pedicle, to a mucous surface. These polypi are liable to occur in any of the mucous cavities of the body, but are most frequently met with in the nose, where they are sometimes found to occupy the superior and middle meati, but most frequently have their location in the inferior, attached to the external side of the nares. They are of many varieties, the principal of which are the hard, which are commonly of a malignant character;—the soft or gelatinous polypi, which are about the consistence of an oyster, are usually benign,—and another variety whose consistence is between that of the fibrous and gelatinous, which is somewhat of a malignant nature. All these, however, give rise to the same symptoms, which are, difficulty in breathing and diminished sense of smell, and are only to be distinguished by examination, which is always necessary before attempting their removal, as their nature should vary the means adopted for the purpose. This may be done very readily by using a pair of polypus, or ordinary forceps, as a speculum, to distend the nares.

The operation necessary in these cases, must be somewhat varied according to the nature, seat, and direction of the tumour. The ligature is only to be used when, from the nature of the tumour, there is danger that hæmorrhage may supervene,—in these, the ordinary one of wire passed around the base of the tumour is the best. When the tumour takes, as it sometimes does, the direction of the fauces, a pair of forceps bent upon themselves, such as you see before you, which were constructed for the removal of a very large one, some time since, from the posterior nares of a gentleman from Virginia, must be used. These are passed into the pharynx until the point will pass into the nares, when after grasping the tumour firmly, it may be removed with facility by a slight rotary motion of the hand. For ordinary cases, such as the one before us, which is of the gelatinous kind, the ordinary polypus forceps are used; these may be either slightly curved, or quite straight, according to the situation of the tumour or the fancy of the operator, the former being more convenient for the upper meatus as may be seen by the drawing on the rack, which represents the operation on an enlarged scale for the removal both from the fauces and superior meatus. After grasping the tumour firmly with the forceps, you see I do not attempt to pull it straight out, as this would probably only bring away a shred, but by causing the forceps to revolve, it is twisted off at its base and brought away entire. You may often be embarrassed, after having promised the patient immediate releif,—first by bringing away only a small shred of the tumour in your forceps, and therefore having to repeat the operation over and over again; and secondly by discovering on the removal of the prominent one, the existence of smaller ones, sometimes to the number of 15 or 20 as I have seen. It is always proper after the removal of one of these gelatinous polypi, to sweep out the meatus as it were, and clear away any remaining obstruction. This may be done very effectually by means of a Belloque's sound passed through the nose into the mouth, then attaching to the loop, which should always be affixed to the end of the instrument, a piece of strong thread, to the middle of which a piece of sponge or lint is firmly tied,—removing the sound, bringing with it one end of the thread to which the sponge is fixed, and finally the sponge itself, which sweeps out every obstruction. This is also a very effectual means of plugging the posterior nares in cases of obstinate epistaxis; a little ingenuity only being required to supply the place of the sound, where it is not at hand.

As these polypi are very apt to recur, it is always best to inform the patient of the fact, and direct something to be used in the way of a preventive.

For this purpose the Pulv. Rhei has been much used and highly spoken of, (to be used as a snuff,) but I have used, in the same way, an impalpable powder of the Sanguinaria Canadensis or Blood Root, with more success; the patient will therefore furnish herself with a snuff box and use it habitually for a time.

Case IV.—Is also one of very common occurrence, and although not presenting a very formidable appearance, often proves very troublesome to the patient. This consists, as may be seen, of a tumour of the lower eyelid. These may be of various kinds; sometimes seated between the cartilage and the skin, filled with hair or cheesy matter; at others involving the cartilage itself, being an enlargement of the Meibomian glands. The latter usually pointing inwards, and sometimes by the pressure against the eye-ball cause the progressive absorption of the cartilage and conjunctiva, finally breaking, and thereby accomplishing a spontaneous cure. This should not be waited for in ordinary cases, as the tissues may become involved to a great extent before its occurrence; but taking the hint from nature, introduce a cataract knife,—lay it open freely, and cause it to heal by granulation. In case the puncture should close, as it sometimes will before the sack is obliterated,—pencilling it with nitrate of silver will be sufficient to effect another opening.

In the ordinary steatomatous (or as some one in Pickwick has called them stay-at-home-with-us) tumours of the lid, occurring in the cellular tissue between the skin and cartilage, which usually appear more superficial,—the whole sack must be removed, for if any part of it be left, it will cause the establishment of a very troublesome fistula, which cannot be healed without a second operation.

In this patient we have an enlargement of one of the glands, which as you see projects into the orbit. You may see now that it is open, the character of the fluid which it contained,—transparent and slightly colored,—by scratching the bottom of the sack with the point of the knife, we will be able to establish sufficient inflamation to cause its obliteration. The smaller one upon the upper lid of the other eye, appears to be of a different character; its consistence is firmer; more of the steatomatous or cheesy nature as you see; the contents have a white granular appearance.

Case V.—Is introduced to exhibit to the class, for particular reasons, the result of the operation performed some weeks since, for the restoration of the right ala of the nose. This, as will be remembered, was accomplished by a subcutaneous section of the tissues, thereby avoiding all scar of the cheek, and presenting, now that it is finished, an appearance as nearly natural as could be desired.

Case VI.—Is a very instructive one, in as much as it presents the character of the deformity resulting from a dislocation of the head of the humerus into the axilla, which ought to be familiar to every one. The squareness of the shoulder, and lengthening of the arm are sufficiently obvious to strike the most superficial observer. The accident occurred three years since, and the patient now presents himself to know if anything can be done for his relief.

He was placed after the occurrence in one of the hospitals of Paris, where every effort at reduction proved unavailing. What should be done with this case? It is one of no light importance to the patient, who is otherwise a powerful and healthy man. Shall we then make any attempt at reduction, or, shall we not rather let it alone? The latter is certainly the proper course;—telling the patient that as he grows older, he will gain the use of it to a great degree, from the formation of a new joint with the head of the bone in its present situation; for, if by any possibility we could get the head back into its proper position, there would be no glenoid cavity for its reception, and therefore impossible to keep it there,—absorption having taken place to an extent sufficient for the obliteration of the natural cavity.

Case VII.—Presents the result of the operation for Aneurism by anastomosis, of last week, upon which it is unnecessary to make any remarks, the tumour having almost disappeared.

Case VIII.—Patient complains of swelling and weakness of the ankle-joint the result of a sprain. The motions of the joint are perfect,—no pain experienced on moving,—his greatest inconvenience being weakness of the joint. The effusion into the cellular tissue here, is doubtless the result of the previous inflammation, and probably of a sub-inflammatory state which still exists. These will best be overcome by local stimulants, which will create a modified action in the part, and thereby break in upon the present morbid condition. We will therefore advise the patient to bathe it freely in cold, or if that disagrees,—warm water, at the same time making use of frictions with some stimulating ointment, which will be probably sufficient to effect a cure. If by any chance these should bring back inflammation, we shall place the limb in a splint; leech the joint &c.

As the time has already expired, I shall not detain the class longer with the other cases,—but merely exhibit in this little girl, who has been brought in, the effects of a blow in producing cataract. Five weeks since she received a blow from a stone, in the eye; active inflammation succeeded, and a cataract as you see has been the result,—for the indication, treatment, &c., I shall refer you to Professor Pancoast, at a succeeding lecture.

In conclusion I will only remark that I expect to sail at an early day for Europe; where I hope to find much that may serve to instruct and interest you at the approaching session, until which time I shall not probably have the pleasure of meeting you again.

